# Balancing the rights of the pre-symptomatic child to be found with the risk of harm to others from the screening process

**DOI:** 10.1038/s41431-024-01689-6

**Published:** 2024-08-30

**Authors:** Anneke Lucassen, Rachel Horton

**Affiliations:** Centre for Personalised Medicine, Nuffield Dept of Medicine, University of Oxford, Old Road Campus, Roosevelt Drive, Oxford, UK

**Keywords:** Ethics, Risk factors, Medical ethics

Newborn screening programmes aim to identify those babies who will go on to develop a condition, so that they can be offered early treatment or intervention to change the course of the disease. Many countries globally have national screening programmes, and although the number and type of conditions included in these programmes varies considerably, most babies will test negative as the conditions screened for are rare. Advances in genomic technology- particularly a reduction in cost and improvement in speed- mean that, in theory, many more rare conditions might now be screened for in the newborn period.

There are major benefits to making reliable diagnoses early where treatments or interventions can alter the course of the disease. Where screening can achieve this, few would disagree that it should be utilised. However, no screening test is perfect and there will always be some incorrect results. False positive or uncertain results can be harmful, as healthy babies might have invasive or harmful tests or treatments which they do not need. Although many might hope that the addition of genomic testing to the screening process will provide both breadth and clarity, in reality these are often in conflict. The more extensive the analysis, the greater the chance of generating uncertainty, and the more we need good research evidence before implementation in public health screening programmes. Consent conversations with parents about genomic screening will need to recognise this greater uncertainty, particularly as it goes against prevailing discourses that promise “*Predictive, preventative, personalised healthcare*” will be provided by genomics (see for example, https://www.dailymail.co.uk/news/article-7654037/DNA-test-save-lives-thousands-children-identifying-inherited-diseases-babies.html).

In this issue of the journal, Knoppers and co-authors [1] make an important point: that the complexity of consent conversations should not derail screening programmes that introduce genomics. They highlight that different activities- clinical care, research and public health programmes- require different types of consent, and argue that in the case of public health screening programmes, the right of the asymptomatic, at-risk, child to be found should guide consent requirements in a way that it cannot do in these other settings.

Arguably in a screening programme all children are at some risk, and it is really the pre-symptomatic child- the child who will actually go on to develop the condition- who needs to be identified. The category of ‘asymptomatic at-risk child’ will also include some children who appear at risk but will not in fact go on to develop the condition in question – and these are the children who in fact stand to be most disadvantaged by the introduction of genomic screening. The authors clarify that their novel right only applies “*to situations where the newborn will be truly ‘found’*” but on a technical level it is often not possible to distinguish which child will go on to develop symptoms and which will have ultimately spurious concerns raised around their health.

Nevertheless, the right of a truly pre-symptomatic child to be found is an appealing concept, and the authors anchor this in the UN Convention on the Rights of the Child (1989) which states that the “*best interests of the child shall be a primary consideration*” and that this imposes a legal duty to maximize the potential for children to enjoy this right. A legal obligation to respect children’s actual and future health rights and needs would mean that complexities of consent- and presumably the possibility of parents not providing it because it all sounds too complicated, or terrifying- should not shroud this right. They invoke this right in specific screening settings- those of carefully curated panel tests [panels that sift particular variants from the millions of variants we all have in our entire genetic code] rather than “*the unconstrained use of genomics in first-line newborn screening*”. They argue that a public health ethics framework is most appropriate for consent conversations. This draws on concepts of solidarity, reciprocity, equity and fairness and allows a more appropriate balancing of consent complexities with the right of the asymptomatic, but at-risk, child to be found.

Attributes such as trust (on the part of parents) that a screening programme will deliver something that is in their child’s interests are important where consent alone cannot do all the ethical work. Research into consent for sequencing a person’s complete genetic code (their genome) has demonstrated that many participants could not remember the details of what they consented to. However, they were not concerned by this and trusted that the health professionals who had sought their consent would act in their interests [2]. Such trust is essential to the success of newborn screening programmes, and as Knoppers et al. state, programmes are unlikely to prosper unless there is widespread ‘buy-in.’ Indeed current [largely, but not exclusively, biochemical] screening is frequently portrayed and perceived as routine care for a newborn baby, rather than a choice over which parents might want to deliberate. For most parents, the offer of screening, often in the first hectic days of a baby’s arrival in the world, may barely register. For those who receive for example, an inconclusive result, the experience is different: as White et al. emphasise, newborn screening “*can have far-reaching implications across time, space, and family groups*” [3].

The right of the pre-symptomatic newborn to be found should also be balanced, therefore, with the right of the majority of babies undergoing screening not to be harmed by the process (more than 99% of the screening population will not be truly pre-symptomatic, even where hundreds of genetic conditions are included). Such a balancing act needs to be made in any newborn screening programme, and is the reason that a lot of diagnoses that have long been technically possible, are not included. Indeed, Knoppers et al. situate their argument in a setting where the balance of harms and benefits has already been researched. Herein lies a problem- whilst the technology to delineate entire genetic codes is available and affordable, the evidence that screening programmes require to be implemented is still being, or is about to be, investigated in a range of different research programmes globally [1].

Distinguishing public health, clinical care and research is important, but in practice, clear dividing lines are difficult. One of the largest newborn screening studies to date to offer genomic sequencing as a means to dramatically increase the number of conditions looked for, is at the same time a research study and a public health endeavour [4, 5]. This project will look for some 200 conditions in the newborn period (as opposed to 9 conditions currently on offer in the UK). However, it is only available to parents who will also consent to research on their baby’s whole genome, linked to their health records, throughout their lifetime. Such research is crucial to find out more about the link between genomic variants and disease, but inevitably requires more detailed consent (including requirements set by research governance) than a public health programme where uncertainty and false positives have already been minimised, and where the vast majority will test negative.

Importantly, Knoppers et al. limit their exploration to public health settings where “*variant calling draws on population level knowledge*”, but the stark reality is that such population level knowledge is lacking for many genetic conditions. They may be too rare to have been studied at a population level or the effect of the variants may vary significantly between different ancestral populations. Here it is important to remember that much of our understanding of genetic variants comes from their discovery in those with overt health problems, that is to say that variants have an ascertainment bias because they have been found mainly, or only, in those who already have the associated disease. How well such variants predict future disease is less clear than we once thought and will need much more research before they meet the criteria of a public health screening programme. Even in relatively well studied conditions like cystic fibrosis where genetic screening already happens to a limited extent in some countries, we know that genetic findings in the absence of other abnormalities (such as a positive biochemical screen) lead to many more uncertain results and false positives. There are no first line newborn genome screening programmes for which there is sufficient evidence for implementation in a public health setting alone: all need research to be done first, or run in tandem, before this evidence is available.

The authors’ point that “*if a consent process will undermine the right of asymptomatic at-risk children to be found through newborn screening, then this provides good reason to limit genomic sequencing to a well curated panel undertaken with appropriate consent*” is an important one, but does beg the question- why ask for whole genomes to undertake newborn screening, with all the privacy and environmental concerns this entails, if the same screening could be provided with more limited genetic data [6]?

Invoking a right to be found imposes corresponding duties on others to facilitate that right. We may need to focus more on the right of the healthy child not to be harmed by years of medical interventions without ultimate benefit, than on the duties of a health service to find babies who cannot be clearly delineated. Unless we get that balancing act right we will also undermine the trust that people place in screening programmes.
